# The Granular Retrosplenial Cortex Is Necessary in Male Rats for Object-Location Associative Learning and Memory, But Not Spatial Working Memory or Visual Discrimination and Reversal, in the Touchscreen Operant Chamber

**DOI:** 10.1523/ENEURO.0120-24.2024

**Published:** 2024-06-14

**Authors:** Paul A. S. Sheppard, Charlotte A. Oomen, Timothy J. Bussey, Lisa M. Saksida

**Affiliations:** ^1^Robarts Research Institute, Schulich School of Medicine and Dentistry, Western University, London, ON N6A 5B7, Canada; ^2^Department of Experimental Psychology, University of Cambridge, Cambridge CB2 1TN, United Kingdom; ^3^MRC and Wellcome Trust Behavioural and Clinical Neurosciences Institute, University of Cambridge, Cambridge CB2 1TN, United Kingdom

**Keywords:** associative learning, paired-associates learning, trial-unique nonmatching-to-location, visual discrimination, working memory

## Abstract

The retrosplenial cortex (RSC) is a hub of diverse afferent and efferent projections thought to be involved in associative learning. RSC shows early pathology in mild cognitive impairment and Alzheimer's disease (AD), which impairs associative learning. To understand and develop therapies for diseases such as AD, animal models are essential. Given the importance of human RSC in object-location associative learning and the success of object-location associative paradigms in human studies and in the clinic, it would be of considerable value to establish a translational model of object-location learning for the rodent. For this reason, we sought to test the role of RSC in object-location learning in male rats using the object-location paired-associates learning (PAL) touchscreen task. First, increased cFos immunoreactivity was observed in granular RSC following PAL training when compared with extended pretraining controls. Following this, RSC lesions following PAL acquisition were used to explore the necessity of the RSC in object-location associative learning and memory and two tasks involving only one modality: trial-unique nonmatching-to-location for spatial working memory and pairwise visual discrimination/reversal. RSC lesions impaired both memory for learned paired-associates and learning of new object-location associations but did not affect performance in either the spatial or visual single-modality tasks. These findings provide evidence that RSC is necessary for object-location learning and less so for learning and memory involving the individual modalities therein.

## Significance Statement

Animal models are essential to understand and develop therapies for diseases such as Alzheimer's disease (AD). Given the importance of the human retrosplenial cortex (RSC) in object-location associative learning and the success of these paradigms in human studies and in the clinic, it is of considerable value to establish a translational model of object-location learning for the rodent. We determined that lesions of the RSC in male rats following object-location paired-associates learning (PAL) led to impairments in object-location associative memory and new learning without affecting performance on tasks of the individual modalities (i.e., spatial and visual). These findings further validate the touchscreen PAL test as a viable translational test for modeling diseases, such as AD, in which RSC is compromised.

## Introduction

The retrosplenial cortex (RSC) is regarded as classical association cortex and a “hub” of interconnectivity, with extensive and diverse connectivity to multiple cortical and subcortical brain regions (van Groen and Wyss, 1990, 1992, 2003; Wyass and Van Groen, 1992; Sporns et al., 2007; Sugar et al., 2011; Rolls et al., 2022). Specifically, rich connectivity with the hippocampus, the cortex, and an array of other regions positions RSC to integrate multimodal, converging information (Todd et al., 2019; Castiello et al., 2021). Consistent with this connectivity, RSC has been reported to have a role in associative learning (Todd and Bucci, 2015; Trask et al., 2021) and spatial navigation (Pothuizen et al., 2008; Vann et al., 2009; A. S. Mitchell et al., 2018; Alexander et al., 2023).

RSC undergoes metabolic, structural, and functional connectivity changes early in Alzheimer's disease (AD) and mild cognitive impairment (MCI; Ma et al., 1994; Minoshima et al., 1997; Scahill et al., 2002; Nestor et al., 2003a,b; Buckner et al., 2005; Chételat et al., 2005; Zhou et al., 2008; Pengas et al., 2010; Aggleton et al., 2016; Dillen et al., 2016) and in animal models of these conditions [Helpern et al., 2004; Poirier et al., 2011; but also see Jullienne et al., (2022, 2023)]. Notably, deficits in associative learning are observed early in these conditions (Sahakian et al., 1988, 1993; Fowler et al., 1995, 2002; Swainson et al., 2001; Blackwell et al., 2003; Egerházi et al., 2007; Quenon et al., 2015; Chipi et al., 2022; Maturana et al., 2023), with performance on associative learning tasks being highly associated with global cognition (Hicks et al., 2021). An especially sensitive test of associative learning and memory in detection of preclinical AD and progression from MCI to AD is the object-location paired-associates learning (PAL) test within the Cambridge Neuropsychological Test Automated Battery (CANTAB; Sahakian et al., 1988, 1993; Fowler et al., 1995, 2002; Swainson et al., 2001; Blackwell et al., 2003; De Jager et al., 2005; Egerházi et al., 2007; Chipi et al., 2022). It has been reported that CANTAB PAL, combined with age, gender, and one other test, can predict the conversion from MCI to AD with high sensitivity (>90%) and specificity (>86%; J. Mitchell et al., 2009; Hicks et al., 2021). Notably, functional magnetic resonance imaging has shown activation during CANTAB PAL retrieval (de Rover et al., 2011) coextensive with regions of reduced metabolism in MCI (Nestor et al., 2003a,b) and AD (Minoshima et al., 1997; Nestor et al., 2003b; Buckner et al., 2005) and reduced connectivity (Zhou et al., 2008), gray matter (Chételat et al., 2005), and overall volume (Scahill et al., 2002) in the conversion from MCI to AD.

To understand and develop therapies for diseases affecting cognition, including AD, animal models are essential. Given the importance of the human RSC in object-location learning and the success of object-location associative paradigms in human studies and the clinic, it is of considerable value to establish a translational model of object-location learning for the rodent. Touchscreen methods allow the use of such tasks in rodents that are in their most relevant aspects identical to those used in humans (Bussey et al., 1994, 2008) and, therefore, allow for validated and robust preclinical cognitive assessment of clinically relevant and highly translatable behaviors (Hvoslef-Eide et al., 2016; Palmer et al., 2021; Sullivan et al., 2021). Alongside its clinical applications, rodent PAL has shown translational validity in that it has revealed impairments in associative learning and memory in rodent models of AD (Beraldo et al., 2019; Izumi et al., 2020; Saifullah et al., 2020; Pang et al., 2022; Liu et al., 2024). Therefore, we tested the role and necessity of RSC of male rats in object-location learning using the object-location PAL touchscreen task, as well as whether RSC is needed for single modality (location or object) learning and memory—using trial-unique nonmatching-to-location (TUNL) and pairwise visual discrimination/reversal (PVD/R) touchscreen tasks, respectively—or only multimodal integration and association.

## Materials and Methods

### Animals

For all experiments, male Lister Hooded rats (250 g at the start of the experiment; Experiment 1, *n* = 21; Experiment 2, *n* = 24) were obtained from Harlan. Rats were housed in groups of four and kept under an inverse dark/light cycle [12/12 h, lights on (Zeitgeber Time 0, ZT0) at 19:00 h] with all testing during the dark phase. Animals were food-restricted to 85% of their normal body weight starting 1 week after arrival and maintained throughout the experiments with water available *ad libitum*. All procedures were conducted in accordance with the United Kingdom Animals (Scientific Procedures) Act, 1986.

### Experimental design: overview

**Figure 1.**
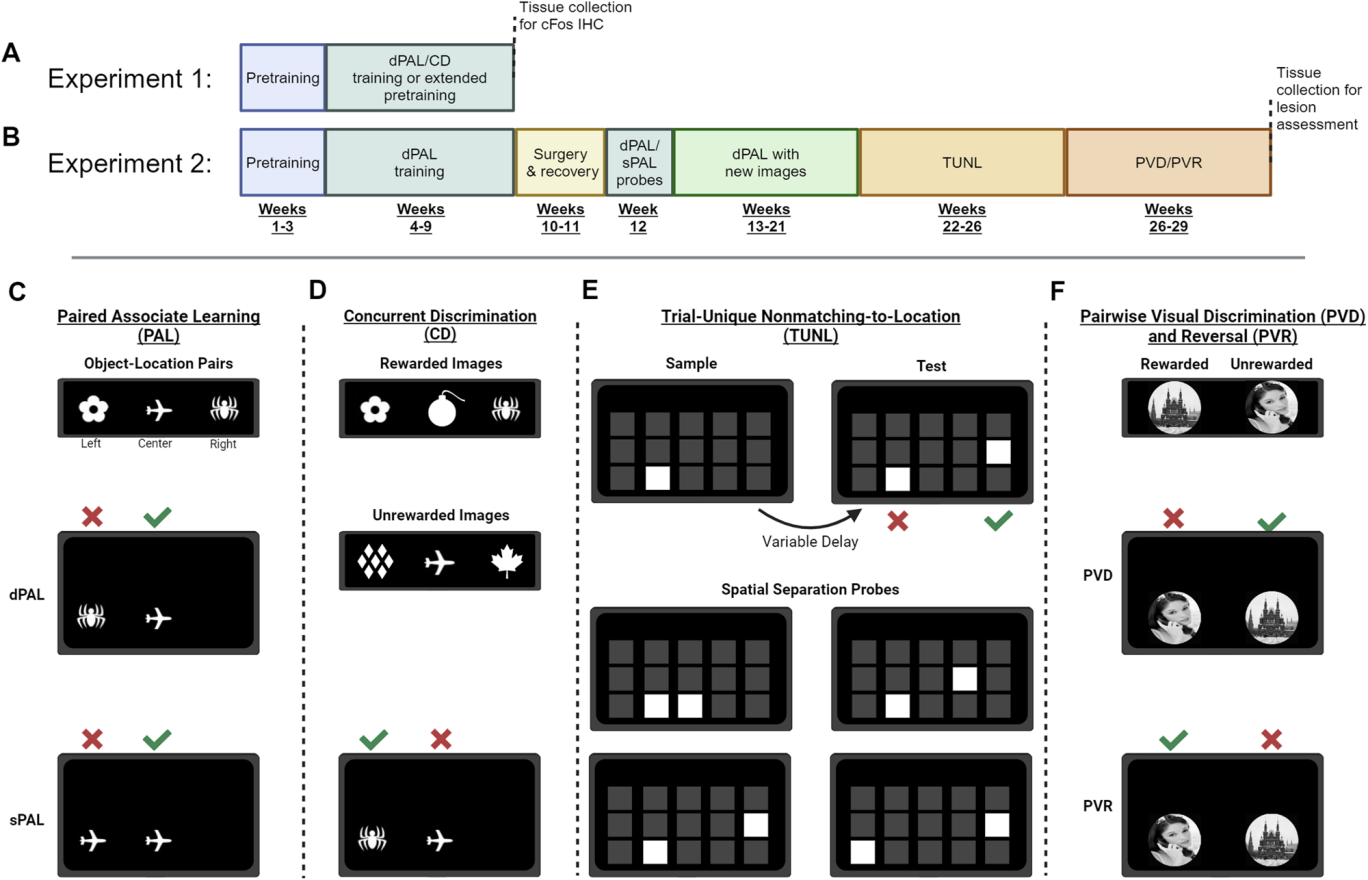
Summary of behavioral tasks and chronological experimental design. ***A***, Timeline for Experiment 1. Rats were food restricted and performed pretraining on Weeks 1–3. Following this, rats were trained on dPAL or CD or performed extended pretraining (Weeks 4–9). Sixty minutes following the final session, rats were killed, and brains were collected for cFos IHC. ***B***, Timeline for Experiment 2. Rats were food restricted, pretrained, and trained on dPAL from Weeks 1 to 9 and then had RSC lesion or sham surgeries (and recovery) during Weeks 10 and 11. They were then tested on dPAL and sPAL (Week 12), dPAL with a new image set (Weeks 13–21), and TUNL (Weeks 22–26), and PVD/R (Weeks 27–29) and then killed for tissue collection and lesion assessment on Week 30. ***C***, PAL requires the rat to respond to the correct image/location pair to obtain reward. There are two versions of this task: dPAL or sPAL. In dPAL two different images are presented with only one in the correct location, whereas in sPAL two identical images are presented with only one in the correct location. ***D***, CD requires the rat to respond to a rewarded image when presented concurrently with an unrewarded image to obtain reward. ***E***, TUNL requires the rat to respond to the illuminated location that is nonmatched to the location illuminated in the sample phase to obtain reward. Various delays are employed to probe spatial working memory, and various separations are employed to vary the load on pattern separation. ***F***, Similar to CD, PVD requires the rat to respond to a rewarded image when presented concurrently with an unrewarded image to obtain reward. Following PVD, the rewarded and unrewarded images were switched (PVR), and rats were required to respond to the previously unrewarded image to obtain reward. Figure made using BioRender.

For the chronological description of experimental procedures, see Figure 1*A,B*. In short, during the first week after arrival, rats were regularly handled and habituated to the animal facility and testing room. After this, pretraining commenced. When rats were able to touch the screen reliably and quickly, they were transferred to the PAL task (*n* = 6), CD (*n* = 7), or extended pretraining (*n* = 8, Experiment 1; tasks outlined below). As soon as a rat had reached a criterion (80% correct, 2 d in a row), its training frequency was reduced from 5 to 7 d a week to one reminder session per week to prevent overtraining. After all animals had reached the criterion, they were subjected to a final three sessions before immediate early gene (IEG) analysis (Experiment 1). Having determined the involvement of the RSC in object-location associative learning, a lesion experiment was conducted in which rats were trained on PAL and excitotoxic lesions targeting the granular RSC (*n* = 12; or sham surgeries, *n* = 11) were performed (one rat was removed for not reaching the criterion after 45 d). Following lesioning, rats recovered from surgery for 1 week and then began a battery of touchscreen tasks (described below) before sacrifice and lesion assessment.

### Immunohistochemistry (IHC)

In Experiment 1, 60 min following the final session of PAL, CD training, or extended pretraining, rats were anesthetized with sodium pentobarbitone (Dolethal, Vetoquinol) and then underwent transcardiac perfusion, with the circulatory system first flushed with 0.01 M phosphate-buffered saline (PBS) following by fixation with 4% paraformaldehyde (PFA) in PBS. Brains were then extracted, postfixed in 4% PFA, and cryoprotected with 20% sucrose in PBS. Brains were coronally sliced using a freezing microtome at a thickness of 30 µm. All stereological quantification procedures described below were performed in every 10th coronal section. Sections were stained for the IEG cFos using the cFos Ab-5 primary antibody (1:1,000, Oncogene), and the biotinylated goat anti-rabbit secondary antibody (1:1,000, Vector) with chromogen development was performed using diaminobenzidine (DAB).

In Experiment 2, following testing, tissues were extracted and fixed (as above). Brains were coronally sliced using a freezing microtome at a thickness of 60 µm. Sections were collected in a phosphate buffer (0.01 M, pH 7.4) and stained for neuronal nuclei (NeuN) using the primary antibody mouse anti-NeuN (1:10,000; Vector) and secondary antibody biotinylated horse anti-mouse (1:200; Vector). Chromogen development was performed using DAB. Nine sections were matched to the rat brain atlas and the extent of lesioning was assessed. Rats with RSC lesions that spared substantial portions of the RSC (*n* = 2) and rats with sham lesions where the infuser damaged the surrounding tissues (*n* = 1) were removed from analyses.

### cFos quantification

The total estimated numbers of cFos-positive cells in all cortical regions (cingulate, prelimbic, infralimbic, orbitofrontal, retrosplenial, and somatosensory) were quantified by systematic random sampling performed with the Stereo Investigator system (MicroBrightField). Stereo Investigator optical fractionator settings (i.e., grid size and counting frame) were different for each region and resulted in at least 250 markers sampled per brain region, per animal. Within the RSC, two markers were distinguished corresponding to the granular and dysgranular layer.

Because of the relatively sparse occurrence of cFos-positive cells in the dorsal and ventral hippocampal subregions (DG, CA3, and CA1 + 2) and striatum subregions (NAc core, NA shell, and CPU), all cells in these regions were counted manually using a Zeiss microscope (200× magnification) and expressed as average number of cells per section.

### Surgeries

Rats were anesthetized with 5% isoflurane and maintained for the duration of surgery at 2% isoflurane (IsoFlo isoflurane, Abbott Laboratories, administered via VetTech Solutions apparatus). Rats were positioned in a stereotaxic frame (David Kopf Instruments), fitted with atraumatic ear bars (Kopf 955), and with a nose bar set to +5 mm. Bilateral injections (10 in total) of 0.2 μl of 0.09 M *N*-methyl-d-aspartic acid (NMDA) in a phosphate buffer (pH = 7.2) were made at AP, −2.3; ML, ±0.6; and DV, −1.6 (10° angle); AP, −3.6; ML, ±0.6; and DV, −1.6 (10° angle); AP, −4.7; ML, ±0.5; DV, −1.6; and AP, −5.8; ML, ±0.8; and DV: −2.0; and AP, −6.7; ML, ±0.8; and DV, −2.0 relative to the skull surface bregma (Paxinos and Watson, 2006) through 10 drilled holes at a rate of 0.1 µl/min using a custom-infusing line connected to a 10 μl Hamilton syringe and the Harvard Instruments “Pump 11” infusion pump. Sham surgeries consisted of the insertion of the infuser into each location with no infusion.

### Behavioral methods

#### Pretraining

All behavioral experiments were carried out in 12 rat touchscreen operant boxes (Campden Instruments; Horner et al., 2013; Mar et al., 2013; Oomen et al., 2013). During pretraining, rats learned to touch illuminated squares on the screen to obtain a food reward. Pretraining consisted of several phases, as described in Table 1 and in published protocols (Horner et al., 2013; Mar et al., 2013; Oomen et al., 2013). Each phase had a criterion such as number of trials completed or a certain percent correct that rats had to meet to be transferred to the next phase of pretraining. Although the length of pretraining varied between animals, the approximate duration of each phase is seen in Table 1. The intertrial interval (ITI) was set to 20 s. A session was complete after a maximum of 90 trials or 60 min elapsed (excepting habituation).

**Table 1.** Summary of touchscreen pretraining

Phase	Description	Criterion	Sessions to criterion (approx.)
Habituation	Rats are put in the touchscreen box, which is powered on, but inactive. Five reward pellets are placed in the reward tray	Eat pellets	1
Initial touch	1 of 3 stimulus locations is randomly illuminated (white square) for 20 s. Disappearance of the stimulus coincides with a tone and delivery of 1 reward pellet (tray light on). Touching the screen (=correct response) results in immediate reward delivery of 3 pellets	None	Set to 1
Must touch	1 of 3 stimulus locations is randomly illuminated and rats are required to touch the screen to get a reward pellet	90 trials	3
Must initiate	Similar to “must touch,” but now rats have to “initiate” trials. At the end of the ITI, the tray light turns on which is a sign for the animal to make a nose poke into the tray which activates the stimuli on the screen	90 trials	2
Punish incorrect	Similar to “must initiate,” but during this phase, rats are punished for touching nonilluminated parts of the screen during trial with a “time-out” of 5 s during which the house light is illuminated	90 trials; 80% correct 2 d in a row	5

Adapted from Oomen et al., (2013).

Following pretraining, rats were trained on touchscreen cognitive tasks. In Experiment 1 (Fig. 1*A*), rats were trained on PAL or concurrent discrimination (CD) or were maintained on pretraining and were then perfused for IEG IHC. In Experiment 2 (Fig. 1*B*), rats were trained on PAL and then underwent RSC or sham lesions prior to probe testing and subsequent touchscreen training and testing.

#### PAL task

The touchscreen PAL task (Fig. 1*C*) was performed as described in Horner et al. (2013). In brief, following trial initiation, images would appear in two of three locations on the touchscreen. Rats had to learn that each image (spider, airplane, and flower) was associated with one correct and rewarded location on the touchscreen, with only one image per trial being in the correct location. A correct response (S+) resulted in reward delivery, whereas an incorrect response (S−) resulted in a 5 s timeout with the house light turned on. Rats were trained in the different PAL (dPAL) version of the task in which the S+ and S− are different images from the three-image set.

In Experiment 2, following recovery from surgery, rats underwent three dPAL probe sessions over 3 d using the same stimuli as in training to assess memory for the learned rule. Following this, they performed a same PAL (sPAL) probe in which two of the same stimuli were presented, one in the rewarded location (S+) and one in an unrewarded location (S−) to test whether animals were using strategies alternative to object-location associative learning such as a the conditional rule of the type; “if the stimulus display is equal to *X*, choose left; if *Y*, choose right” (Horner et al., 2013). Subsequently, rats were trained on dPAL using a novel image set (diamonds, bomb, and maple leaf) to assess relearning the object-location associative learning task.

In both the initial learning of dPAL and learning the new image set in Experiment 2, a criterion was set as completion of 100 trials within 60 min, with a score of 80% or greater correct for 2 consecutive days.

#### CD task

The CD task (Fig. 1*D*) is similar to PAL in that pairs of stimuli (one S+ and one S−) are presented in two of three locations. Rats learn to respond to a set of S+ stimuli (flower, spider, and bomb) and not to S− stimuli (airplane, diamonds, and maple leaf). Unlike PAL, the S+ is rewarded regardless of location. This task requires object discrimination (rewarded vs nonrewarded) but not the spatial or object-in-location components of PAL. Rats were run on this paradigm until a criterion was reached (90 trials, 80% correct for 2 consecutive days) and then maintained with weekly reminder sessions to avoid overtraining until all rats had reached the criterion.

#### TUNL task

The TUNL task (Fig. 1*E*) was performed as described in Talpos et al. (2010) and Oomen et al. (2013). Each TUNL trial consisted of a sample and a choice phase. During the sample, the rat was required to nose poke an illuminated touchscreen window and then return to the reward magazine to initiate the choice phase. During the choice phase, following a delay, the window from the sample phase was illuminated as well as a second window, with a nose poke to the latter, and the novel (nonmatching) window as a correct response. The delay between the sample and the choice (0.5, 3, 6, and 9 s) and the separation between the windows (number of blank windows between them, 0–3) during the choice were varied to vary the working memory and spatial discrimination difficulty, respectively. In the present study, rats were trained with a 2 s delay on separations 0–2. A criterion was set at 80% correct on separation 2 for 2 consecutive days.

#### PVD/R task

The PVD task (and subsequent rule reversal; Fig. 1*F*) was performed as in Horner et al. (2013). In this task, rats were trained to discriminate between two concurrently presented visual stimuli (face or building, as in Winters et al. (2010), counterbalanced), with the spatial location randomized across each trial. As in PAL, a correct response (S+) was met with a tone and pellets were dispensed. An incorrect response (S−) resulted in a 5 s time-out during which the house light was illuminated. Following this, a correction trial was initiated with the same stimulus configuration as the preceding incorrect trial. This was repeated until the rat made a correct response. Correction trials were quantified but were not included in the session trial limit (100) nor in the session accuracy score. In the present study, rats performed the PVD task for 10 sessions. Following this, the PVR was initiated, and the formerly rewarded stimulus became nonrewarded and vice versa. Rats performed eight sessions with this new rule.

#### Statistical analyses

Statistical analyses were performed using GraphPad Prism (Version 9.1.0). The IEG expression was assessed with one-way ANOVAs with Tukey post hocs and the behavioral condition (dPAL, CD, or control) as the between-subjects factor, with outliers removed using the robust regression and outlier removal (ROUT) method (*Q* = 1%) and Brown–Forsythe ANOVAs employed when standard deviations were unequal. Two-way ANOVAs were performed for data with multiple sessions, with session and condition (i.e., lesion) as factors. To control false discovery rate, Benjamini, Krieger, and Yekutieli post hocs were performed using the two-stage setup method and *Q* set at 0.05. Other pairwise comparisons were performed using unpaired *t* tests. To assess whether RSC lesions affect preservative responding in our tasks, a perseveration index was calculated as (number of correction trials) / (number of incorrect responses) and analyzed as described above. Two-tailed statistical significance was set at *p* < 0.05.

## Results

### PAL testing increased the number of cFos immunoreactive cells in the RSC

**Figure 2.**
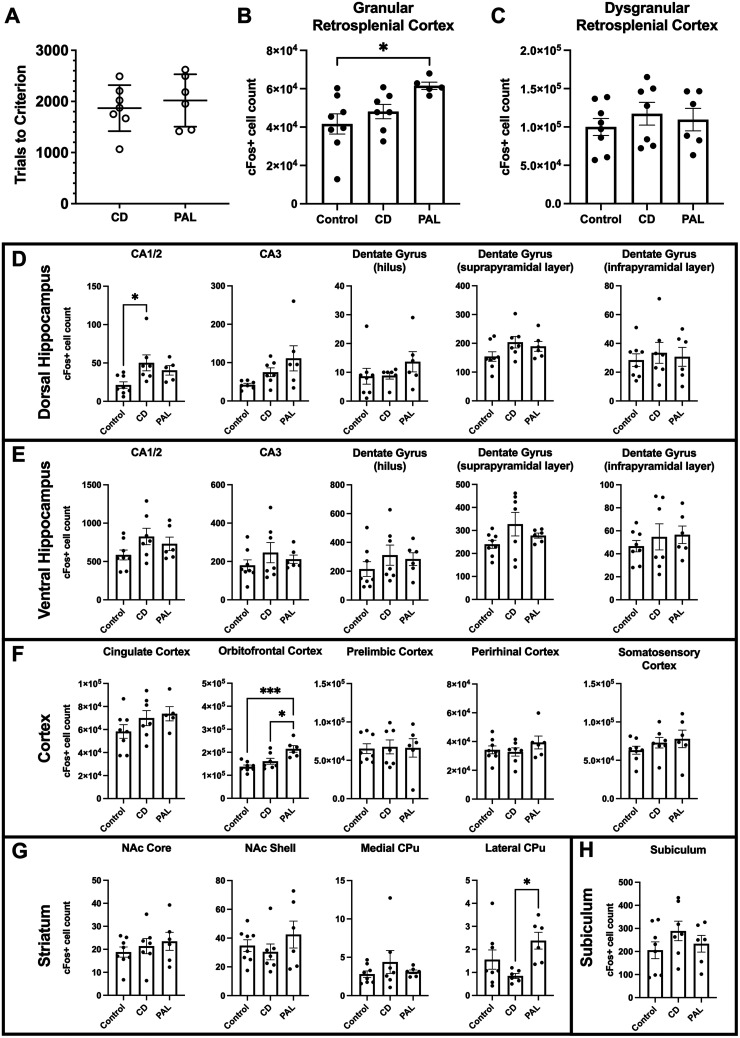
cFos expression in the male rat brain following touchscreen training. ***A***, Trials to criterion did not differ between the CD and dPAL tasks. ***B***, Rats that performed the dPAL task had more cFos+ cells in the granular RSC than extended pretraining controls. ***C***, No difference in cFos+ cell counts in the dysgranular RSC. ***D***, Rats that performed the CD task had more cFos+ cells in the dorsal CA1/2 than extended learning controls. No difference in cFos+ cell counts in other dorsal hippocampal regions. ***E***, No difference in cFos+ cell counts in the ventral hippocampal regions. ***F***, Training on PAL increased the number of cFos+ cells in the orbitofrontal cortex as compared with training in CD and extended training controls. No difference in cFos+ cell counts in other cortical regions. ***G***, Training on PAL increased the number of cFos+ cells in the lateral caudate–putamen as compared with training in CD. No difference in cFos+ cell counts in other striatal regions. ***H***, No difference in cFos+ cell counts in the subiculum. *p < 0.05, ***p < 0.001. Data represented as mean ± SD in (***A***) and mean ± SEM in (***B–H***).

To first determine the neuroanatomical regions active during associative learning performance, rats performed either the dPAL or CD task or were maintained on the final touchscreen pretraining stage (control). Critically, the number of trials required to reach a criterion in dPAL and CD was similar (*p* = 0.583; Fig. 2*A*), so results of the IEG analysis reflect differences in the task requirements (i.e., associative vs nonassociative memory) and not the effects of the training duration. The expression of cFos differed between learning conditions in the granular RSC [*F*_(2,17) _= 4.693; *η*^2^ = 0.35; *p* = 0.0238] with the cFos expression following dPAL significantly greater than in extended pretraining control rats (Cohen's *d* = 1.763; *p* < 0.0186; Fig. 2*B*), but did not reach statistical significance in rats that performed CD (*p* = 0.140). The dorsal CA1/2 [*F*_(2,17) _= 4.366; *η*^2^ = 0.339; *p* = 0.0295; Fig. 2*D*], orbitofrontal cortex [*F*_(2,18) _= 11.55; *η*^2^ = 0.562; *p* = 0.0006; Fig. 2*F*), and lateral caudate–putamen [*F*_(2,17) _= 4.249; *η*^2^ = 0.333; *p* = 0.0319; Fig. 2*G*] showed changes in the cFos expression following learning. In the dorsal CA1/2, CD learning resulted in greater cFos expression than in control rats (Cohen's *d* = 1.362; *p* = 0.0256; Fig. 2*D*). In the orbitofrontal cortex, dPAL learning resulted in greater cFos expression than CD (Cohen's *d* = 1.544; *p* = 0.0128; Fig. 2*F*) and control rats (Cohen's *d* = 2.677; *p* = 0.0004; Fig. 2*F*). In the lateral caudate–putamen, dPAL learning resulted in greater cFos expression than CD (Cohen's *d* = 2.326; *p* = 0.025; Fig. 2*G*). The dysgranular RSC did not exhibit changes in the cFos expression (*p* = 0.647; Fig. 2*C*) nor did other dorsal (*p*s > 0.0566; Fig. 2*D*) or ventral (*p*s > 0.150; Fig. 2*E*) hippocampal, cortical (*p*s > 0.221; Fig. 2*F*), or striatal (*p*s > 0.429; Fig. 2*G*) regions, nor the subiculum (*p* = 0.306; Fig. 2*H*).

### RSC lesions impaired object-location associative memory and learning of new associations

**Figure 3.**
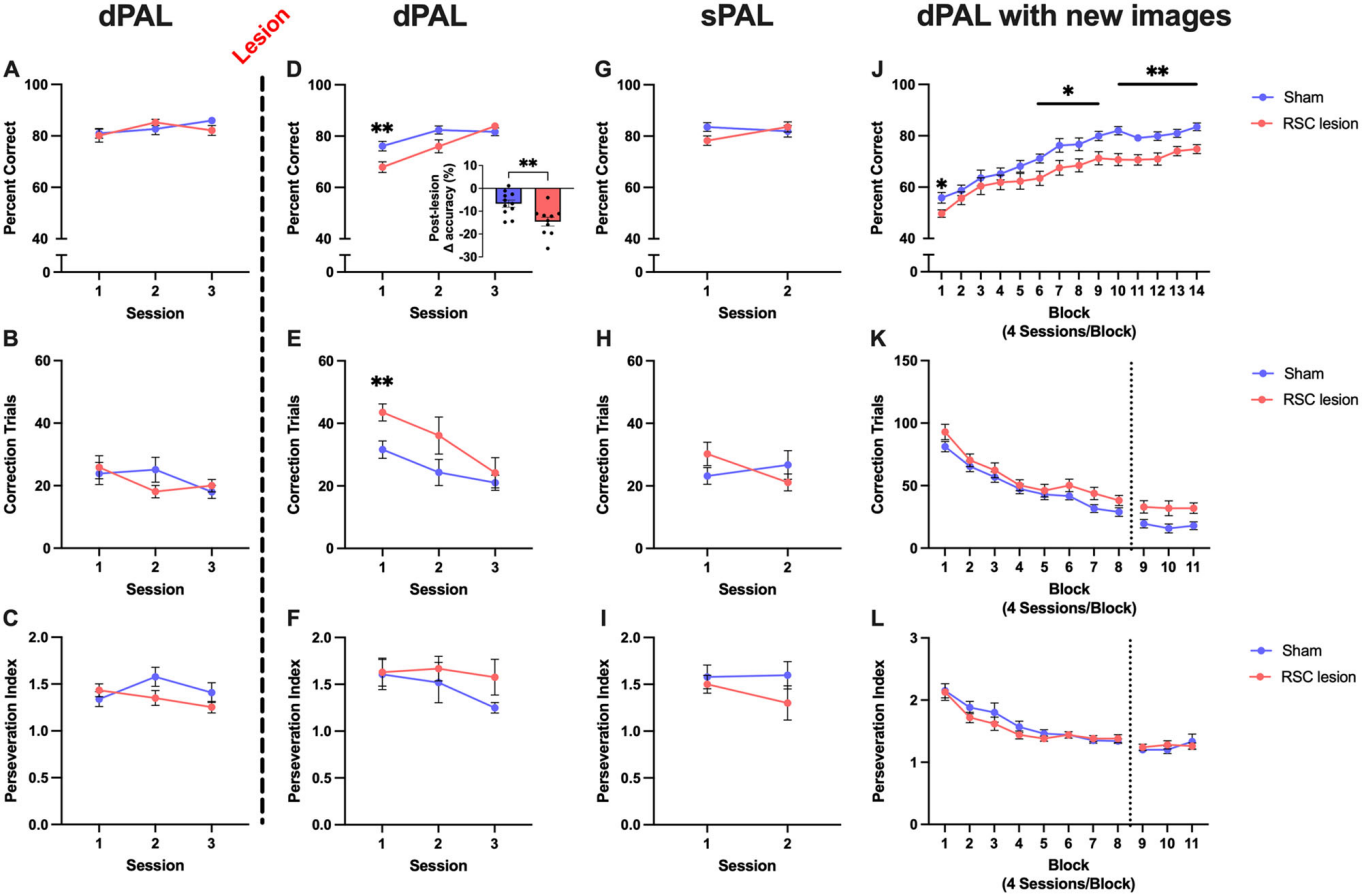
RSC lesion impairs memory for and new learning of object-location associations. Dashed line separates pre- and postlesion data. ***A–C***, Prelesion dPAL performance did not differ between lesion groups. ***D***, Following RSC lesion (see Fig. 3-1 for extent of lesions), rats made fewer correct responses on Session 1 of dPAL than did sham-lesioned rats. Both groups showed a drop in performance compared with prelesion performance (inset), but this drop was greater in the RSC lesion group. ***E***, Following RSC lesion, rats performed more correction trials on Session 1 of dPAL than did sham-lesioned rats. ***F***, RSC lesion did not alter preservative responding in dPAL. ***G–I***, There were no group differences in sPAL performance. ***J***, RSC-lesioned rats were impaired in acquisition of a novel set of object-location associations in dPAL. ***K***,***L***, There were no group differences in correction trials or perseveration during acquisition of a novel set of object-location associations in dPAL. The dotted line in these panels indicates a shift from *n* = 20 to *n* = 8 as a result of data loss. *p < 0.05, **p < 0.01. Data represented as mean ± SEM.

We next performed a lesion study in a novel cohort of rats in which, following dPAL acquisition, rats received either an excitotoxic lesion (using NMDA) or a sham lesion aimed at the granular RSC (postmortem lesion assessment: Extended Data Fig. 3-1). Prelesion acquisition of dPAL did not differ between RSC- and sham-lesioned rats (*p* = 0.548, data not shown), nor did performance on the final three sessions (baseline probes; accuracy, *p* = 0.696, Fig. 3*A*; correction trials, *p* = 0.719, Fig. 3*B*; perseveration index, *p* = 0.218, Fig. 3*C*). Both RSC- and sham-lesioned groups dropped in accuracy following surgery and recovery, but the drop in performance from prelesion baseline dPAL probes to the first postlesion dPAL probe was significantly greater in the RSC lesion group (*t* = 3.202; df = 19; Cohen's *d* = 1.393; *p* = 0.0047; Fig. 3*D*, inset). RSC-lesioned rats had significantly lower accuracy in the first postlesion dPAL probe [Cohen's *d* = 1.321, *p* = 0.0086; session by condition interaction: *F*_(2,36) _= 7.503, *η*^2^ = 0.0854, *p* = 0.0019, Fig. 3*D*; no main effects of lesion on accuracy (*p* = 0.0566, Fig. 3*D*) or (correction trials: *p* = 0.0766, Fig. 3*E*)]. There were no effects on perseveration (*p*s > 0.177; Fig. 3*F*). While there was no significant session by condition interaction in correction trials (*p* = 0.1847; Fig. 3*E*) but there was a significant difference in accuracy between groups in the first session, a Tukey-corrected pairwise comparison (Howell, 1987) within this session was performed and revealed that RSC-lesioned rats performed significantly more correction trials (Cohen's *d* = 1.378; *p* = 0.0064; Fig. 3*E*). Groups performed similarly by the third dPAL probe (accuracy, *p* = 0.270; correction trials, *p* = 0.564; Fig. 3*D,E*). Performance on sPAL probes was not significantly different in RSC-lesioned rats and controls (accuracy, *p* = 0.4811; correction trials, *p* = 0.8609; perseveration index, *p* = 0.214; Fig. 3*G–I*); however, there was a significant session by lesion interaction in accuracy [*F*_(1,17) _= 8.003; *p* = 0.012; Fig. 3*G*] and correction trials [*F*_(1,17) _= 4.517; *p* = 0.049; Fig. 3*H*], with accuracy increasing between Sessions 1 and 2 in RSC-lesioned rats only (Cohen's *d* = 0.932; *p* = 0.0085; Fig. 3*G*; note: there were incomplete data for one RSC-lesioned rat, so it was removed from the sPAL analysis). To test the involvement of the RSC in learning new object-location associations, rats performed the dPAL task a second time with a novel set of images. RSC-lesioned rats were significantly impaired on acquisition of a new set of dPAL image-location pairs [*F*_(1,18) _= 6.404; *η*^2^ = 0.102; *p* = 0.0209; Fig. 3*J*] with impairments being most prominent in the latter half of testing [accuracy: session by lesion interaction, *F*_(13,234) _= 2.227, *p* = 0.0092; blocks 6–9, Cohen's *d*s > 1.002, *p*s < 0.038; blocks 10–14, Cohen's *d*s > 1.3, *p*s < 0.0096; Fig. 3*J*]. Due to the loss of data, the correction trials were complete only up to block 8 with all animals (*n* = 20), with only *n* = 8 for blocks 9–11. While the analysis of these data indicates no effect of lesion (all animals through block 8, *p* = 0.221; eight animals through block 11, *p* = 0.423; Fig. 3*K*), we cannot conclusively ascertain that there was no effect of lesion on correction trials (or perseveration: all animals through block 8, *p* = 0.571; eight animals through block 11, *p* = 0.42; Fig. 3*L*) in the new dPAL learning. Overall, these data indicate that RSC lesions impaired memory of pre-lesion dPAL and learning of a new set of objects in dPAL.

10.1523/ENEURO.0120-24.2024.f3-1 Figure 3-1 **Extent of retrosplenial cortex lesions.** A) Representation of the maximum (light grey) and minimum (dark grey) lesioned regions. Adapted from (Paxinos and Watson, 2006). B) Representative images of RSC lesion (upper) and sham lesion (lower) tissue. Lesions were highly specific to the granular RSC leaving the dysgranular RSC largely spared. Download Figure 3-1, TIF file.

Similar to the correction trial and perseveration index data above, latency data for PAL, as well as TUNL and PVD/R, were lost and were thus absent from this study. It remains possible that RSC lesions could result in changes in response and/or reward collection latencies during testing; however, there is no evidence that motoric ability or motivation requires RSC, and furthermore, there were no differences in the total number of trials in any of the tasks (all *p*s > 0.05), indicating that animals were sufficiently motorically competent and motivated to complete the tasks as required.

### RSC lesions did not affect learning and memory in single modalities

Having observed that RSC lesions impaired learning and memory in PAL, in which visual and spatial information were combined, we subsequently tested RSC-lesioned and control rats on the TUNL test of spatial working memory and the PVD/R task for visual discrimination learning and reversal, which tests learning and memory for stimuli in a single modality, either spatial or visual, respectively.

#### Spatial: TUNL

**Figure 4.**
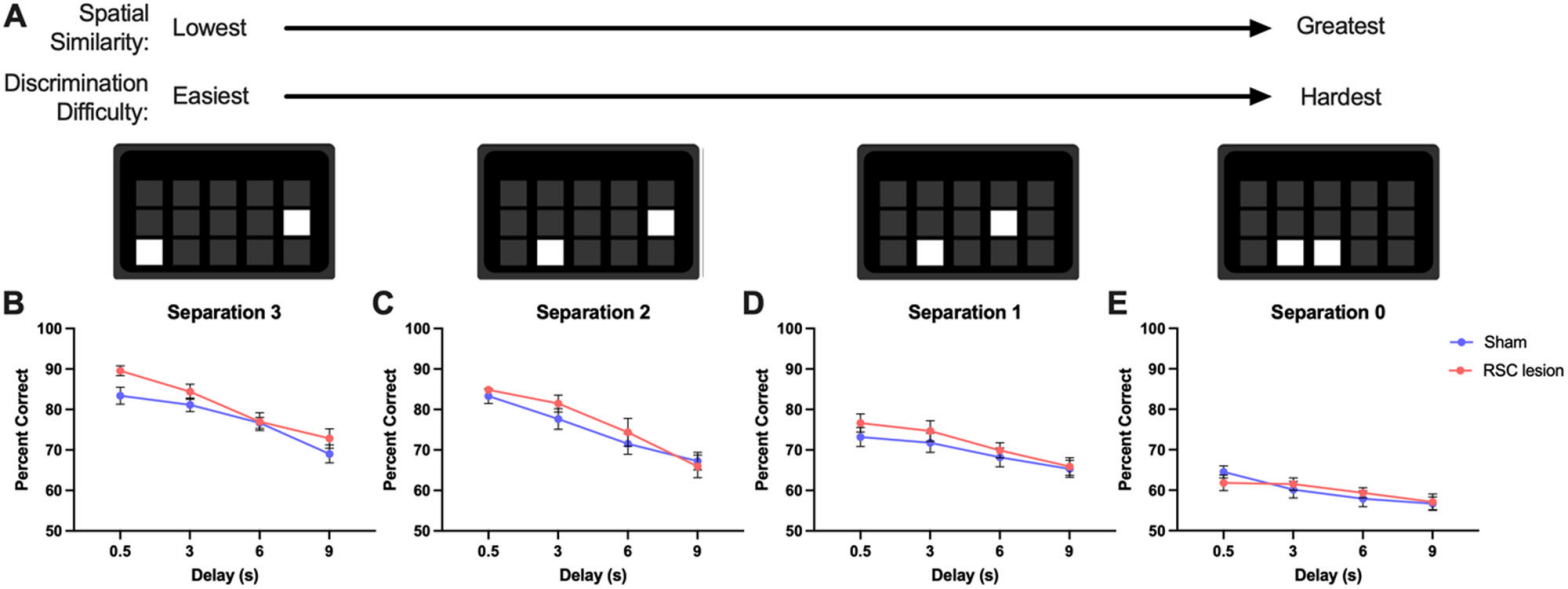
RSC lesion does not affect spatial working memory. ***A***, Examples of response window separations during TUNL testing. As spatial similarity of the response windows increases, so too does the difficulty of discrimination. ***B–E***, There were no group differences in percent correct responses at any separation level or delay. Data represented as mean ± SEM.

Lesioned and control rats did not differ in the rate of learning (*p* = 0.873, not shown). During probe trials (Fig. 4*A* for schematic), lesion groups performed similar to one another across different spatial separations and different delays (*p*s > 0.113; Fig. 4*B–E*). Thus, spatial working memory was unaffected by granular RSC lesions, and the impairments in object-location associative learning were likely not due to deficits in spatial processing alone.

#### Visual: PVD/R

**Figure 5.**
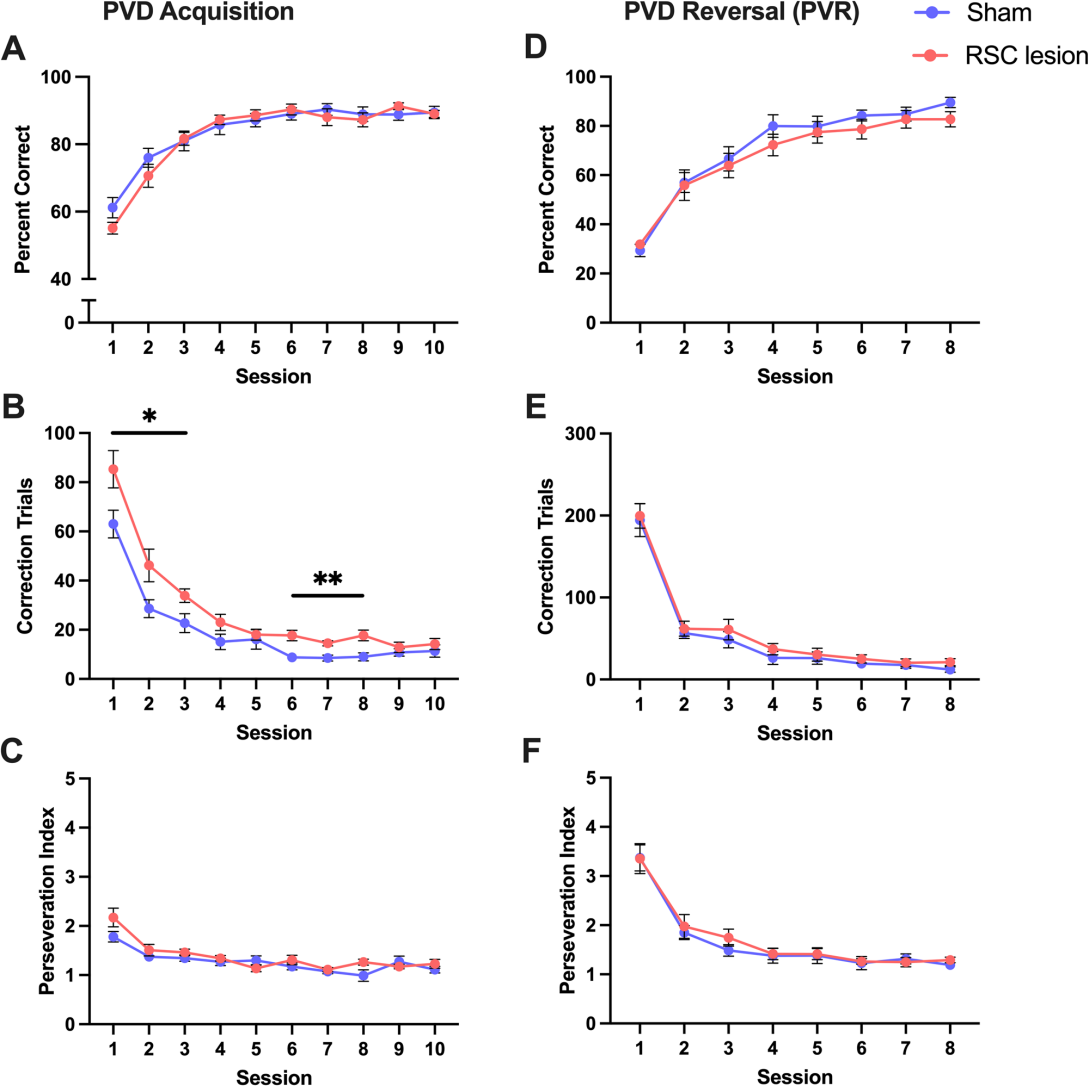
RSC lesion does not affect visual discrimination learning or reversal. ***A***, There were no group differences in percent correct responses across PVD acquisition. ***B***, RSC-lesioned rats performed more correction trials than sham-lesioned rats in PVD acquisition. ***C***, There were no group differences in perseveration across PVD acquisition. ***D–F***, There were no group differences in percent correct responses, number of correction trials, or perseveration following rule reversal (PVR). *p < 0.05, **p < 0.01. Data represented as mean ± SEM.

Both groups learned the PVD task at a similar rate as accuracy did not differ between groups (*p* = 0.726; Fig. 5*A*). However, RSC-lesioned rats performed more correction trials than sham rats [*F*_(1,18) _= 10.42; *η*^2^ = 0.0413; *p* = 0.0047; Fig. 5*B*], with increases in correction trials observed in Sessions 1–3 (Cohen's *d*s > 1.04; *p*s < 0.036) and 6–8 [Cohen's *d*s > 1.45; *p*s < 0.0049; session by lesion interaction, *F*_(9,162) _= 2.415; *η*^2^ = 0.021; *p* = 0.0134; Fig. 5*B*). This increase in correction trials was not sufficient to produce an increase in perseverative index in RSC-lesioned rats (lesion, 0.0872; session by lesion, 0.0647; Fig. 5*C*). Performance following rule reversal did not differ between groups (accuracy, *p* = 0.464, Fig. 5*D*; correction trials, *p* = 0.388, Fig. 5*E*; perseveration index, *p* = 0.649, Fig. 5*F*). In summary, RSC-lesioned rats performed more correction trials when learning the visual discrimination task, but this did not affect the accuracy. Furthermore, they showed no impairments in reversal learning.

## Discussion

The use of animal models and the development of appropriate tasks are essential to understanding and developing effective therapies for diseases affecting cognition, including AD. Given the importance of human RSC in object-location learning and the success of object-location associative paradigms in human studies and in the clinic, it is of considerable value to use translational models of object-location learning for the rodent. For this reason, we sought to test the role of RSC in object-location learning using the object-location PAL touchscreen task for rats.

First, we found that the rat RSC showed increased cFos expression (a proxy for neural activation) following object-location PAL, suggesting it is actively recruited during the performance of the task. The lateral caudate–putamen and orbitofrontal cortex also exhibited increased cFos expression during PAL performance. PAL has been shown to be sensitive to lesions of the dorsal striatum in the mouse (Delotterie et al., 2015). Both the orbitofrontal cortex (Schoenbaum and Roesch, 2005; Hall-McMaster et al., 2017) and striatum (Yin and Knowlton, 2004; Liljeholm and O’Doherty, 2012; Garr, 2017; Stelly et al., 2020; Avvisati et al., 2024) have been implicated in associative learning and memory more generally. There is some evidence of functional connectivity between the RSC and both regions (Monko and Heilbronner, 2021), but whether the observed concurrent activation of these regions during PAL results from direct interactions between the regions remains to be determined. Interestingly, despite many reports demonstrating hippocampal function as essential to rodent PAL (Talpos et al., 2009; C. H. Kim et al., 2015; Delotterie et al., 2015; M. Kim et al., 2016; Al-Onaizi et al., 2017), the only increase in the cFos expression we observed was in rats that performed CD compared with extended pretraining controls. This lack of clear hippocampal involvement in PAL may be a result of the extent of training on PAL prior to tissue collection. One model of the RSC–hippocampal interaction during learning (Miller et al., 2014) places the RSC in a dual role: (1) the RSC outputs to the hippocampus information about cues and context and (2) is the target of the hippocampal output during systems-level consolidation. In this way, following extensive associative learning, it is possible that the performance of a task will have significantly reduced hippocampal involvement or even become hippocampal independent, as has been previously demonstrated in a flavor-location paired-associates task (Tse et al., 2007).

Damage to the RSC following dPAL acquisition transiently impaired memory for the task, and learning of new object-location associations in the PAL task was impaired, indicating a role for the RSC in both learning of and memory for object-location associations. This role appears to be relatively selective, as performance in tasks requiring only spatial or visual learning and memory, as measured by the TUNL and PVD/R tests, was unaffected. These data collectively suggest that, consistent with its patterns of connectivity, the RSC is required for the integration of spatial and visual information but is less important for spatial or visual information processing on their own. The RSC lesions were still present and complete following the end of the testing battery (see Extended Data Fig. 3-1). While in some cases compensatory mechanisms can lead to recovery of function following brain damage, the lesions in the present study were still functionally effective through PVD testing, as rats with RSC lesions performed significantly more correction trials than controls during this task.

There is a growing literature identifying the RSC as the associative cortex important for multimodal integration (Bussey et al., 1996, 1997; Robinson et al., 2011, 2014; Fournier et al., 2020; McElroy et al., 2024) as well as in other types of associative learning (Keene and Bucci, 2008; St-Laurent et al., 2009; Kwapis et al., 2014, 2015; Todd et al., 2016; Katche and Medina, 2017; Sigwald et al., 2019; Yamawaki et al., 2019; Fournier et al., 2019a,b). In agreement with the findings of the present study, it has been suggested that the RSC is especially important for the integration of multiple stimuli, rather than learning and memory for simple associations (Todd and Bucci, 2015; Todd et al., 2019; de Landeta et al., 2020). It has been suggested that in reports where impairment of the RSC function appears to affect singular modalities (de Landeta et al., 2020; Fournier et al., 2020), responses could be influenced by context, which is often characterized by the integration of multiple stimuli (Todd et al., 2019). Critically, the convergence of these data from markedly different associative learning tasks—some appetitive, some aversive, various stimulus modalities throughout—is highly suggestive of a consistent role of the RSC in associative learning. With respect to testing object-in-place associative learning and memory, these different approaches confer different advantages. Touchscreen PAL confers a number of advantages, including a high degree of standardization, robust translational and construct validity, high-throughput data collection, and minimal experimenter involvement during testing. Spontaneous tasks in open fields confer different advantages; for example, usable data gathered from a low number of trials, and a sample-delay-choice structure that allows for the isolation of memory processes such as encoding, consolidation, and retrieval (such as in McElroy et al., 2024).

In the present experiment, since the results of the IEG analysis following PAL revealed an activity in the granular RSC, our lesions targeted the granular RSC and almost completely spared the dysgranular RSC. It has been suggested that the granular and dysgranular divisions of the RSC have shared as well as combined properties and functions (Aggleton et al., 2021). Interestingly, lesioning of the dysgranular RSC shifted rats toward using an egocentric rather than allocentric strategy to solve a spatial working memory task in the radial arm maze (Vann and Aggleton, 2005). The dysgranular RSC appears to be more involved in the processing of distal visual cues in support of spatial working memory (Pothuizen et al., 2009). Notably, lesioning of the granular RSC impaired spatial working memory similar to complete RSC lesions, but granular RSC-lesioned rats were significantly worse than complete RSC-lesioned rats in a spontaneous alternation task that eliminated the use of intramaze cues and thus relied on extramaze cues that were unreliable in half of the trials (Pothuizen et al., 2010). Extensive lesioning of the rostrocaudal RSC impaired tests of allocentric memory but not egocentric discrimination, with clear impairments in spontaneous object-in-place discrimination (Vann and Aggleton, 2002). Caudal-specific RSC lesions mimic these deficits in allocentric memory but with attenuated effects (Vann et al., 2003), supported by later work investigating lesion size on navigation and spatial working memory (Vann and Aggleton, 2004). When comparing granular and dysgranular RSC, overall, the granular RSC receives much hippocampal and parahippocampal information (spatial and contextual), whereas the dygranular RSC receives visual input, with extensive cross talk between the regions (Aggleton et al., 2021). As considerable research has shown the hippocampus to be involved in rodent PAL (Talpos et al., 2009; C. H. Kim et al., 2015; Delotterie et al., 2015; M. Kim et al., 2016; Al-Onaizi et al., 2017), it is not surprising that the granular RSC specifically would be involved in touchscreen PAL, with less involvement of the dysgranular RSC as the touchscreen chamber creates an overall less visually rich environment than in many conventional tasks.

While object-location associative learning was impaired by lesions of the granular RSC, spatial working memory and visual discrimination were spared. The lack of effect on visual learning was anticipated, as it is the dysgranular, not the granular, RSC that is most involved in processing visual inputs (van Groen and Wyss, 1992; Aggleton et al., 2021) and the RSC may only be required for the integration of stimuli with a spatial component (Nelson et al., 2018). The lack of effect of granular RSC lesions on spatial learning and memory may at first appear to conflict with the existing literature (reviewed in Aggleton et al., 2021). However, a closer examination of the types of spatial learning and memory paradigms used reveals a possible explanation: rats run in radial arm or Morris water mazes or on spontaneous alternation tasks are impaired, whereas they are unimpaired in the operant touchscreen TUNL task. While there are significant differences between these tasks, perhaps the most salient is the relationship between spatial information and local cues within the apparatus. In the touchscreen task, there are no distal visual cues of the type typically used in maze tasks; indeed, the available spatial cues with which to delineate spatial locations are part and parcel of the local testing environment. In the classical tests, the distal visual cues and apparatus are clearly separate. This difference could be critical, given the idea that context and its relationship to cue are important in the contribution of the RSC to strategy and performance (Todd et al., 2019). There is previous evidence for neural dissociations on this basis; for example, rats with lesions to the posterior cingulum bundle and adjacent RSC were unimpaired (Aggleton et al., 1995) or had mild improvements (Neave et al., 1996) in performance on delayed nonmatching-to-position in an operant chamber but were impaired in a spatial spontaneous alternation task. Thus, the RSC may be important in spatial tasks requiring the integration of information from distal and local cues, an idea broadly consistent with a role for the RSC in object-location associative learning. In this way, the touchscreens allow for more selective testing of spatial working memory using TUNL by removing or reducing the salience of cue-context or proximal-distal association. However, classical tests are still very useful for the evaluation and understanding of the use of allocentric, egocentric, intramaze, and extramaze cues in task performance as they can be experimentally manipulated and, as such, have contributed much to our evolving understanding of the role of the RSC and its divisions in spatial memory.

One limitation of the present study is the assessment of potential sex differences. As there are known sex differences with regard to prevalence, progression, severity, and even underlying fundamental mechanisms in diseases including AD (Mielke, 2018; Demetrius et al., 2021; Cui et al., 2023) and MCI (Sohn et al., 2018; Williamson et al., 2022) and in age-related cognitive deficits (Febo et al., 2020; Levine et al., 2021), understanding and appreciating the role sex plays in RSC-dependent associative learning is critical. Recent evidence using transient silencing of the anterior RSC in a classical object-location associative memory task has found no sex differences (McElroy et al., 2024). However, there are anatomical and cellular sex differences in the RSC (Guma et al., 2024; Mayne et al., 2024), which could produce sex-specific effects on cognition. Therefore, the present findings using male rats only should not be generalized to females as it cannot be conclusively ascertained whether the observed effects would persist. Future experiments should strive to include both sexes and power their studies such that sex differences can be detected should they exist.

Future studies should also explore the mechanistic roles played by the RSC in associative learning and memory. For instance, as glutamate, acetylcholine, and dopamine neurotransmitter systems have previously been implicated in PAL (Day et al., 2003; Talpos et al., 2009; Bethus et al., 2010; Bartko et al., 2011; Harel et al., 2013; Lins and Howland, 2016), and there is evidence of each of these systems within the RSC (Corcoran et al., 2011; Todd et al., 2019; Vallianatou et al., 2019; de Landeta et al., 2022), it would be advantageous in future studies to explore the neurotransmitters involved in the role of the RSC in associative learning.

In summary, hypometabolism and changes in structure and connectivity in the RSC are established markers of prodromal AD, and a deficit in learning and memory of object-location PAL is a validated clinical behavioral marker of early AD and the progression of MCI to AD. The present results indicate a necessary role for the rat RSC in object-location PAL and memory and further validate the rodent touchscreen PAL test as a translational test for modeling diseases, such as AD, in which the RSC is compromised.

## Synthesis

Reviewing Editor: Mark Laubach, American University Decisions are customarily a result of the Reviewing Editor and the peer reviewers coming together and discussing their recommendations until a consensus is reached. When revisions are invited, a fact-based synthesis statement explaining their decision and outlining what is needed to prepare a revision will be listed below. The following reviewer(s) agreed to reveal their identity: John Howland, Sydney Trask.

Two experts reviewed your manuscript. They found the study compelling but requested some revisions. Please address all points raised in the reviews below. Thank you for sending your manuscript to eNeuro.

Please also consider providing an image for a visual abstract. It helps promote your paper on social media and the eNeuro website.

Reviewer 1

The manuscript provides the results of two experiments to assess the role of rat retrosplenial cortex in three cognitive tasks assessed in touchscreen-equipped operant conditioning chambers. Results indicated increase c-fos expression in granular retrosplenial cortex and orbitofrontal cortex following training of experienced male rats in the paired associates learning (PAL) task. Post-acquisition lesions of retrosplenial cortex also impaired performance of PAL without effects on the trial-unique non-matching-to-location task or a paired discrimination and reversal learning task. Results are considered in the context of rodent models of Alzheimer's disease.

Overall, the manuscript provides some interesting and important data regarding the role of retrosplenial cortex in cognition that will be of interest to behavioral neuroscientists. There are a number of points of concern with the manuscript in its present form. These are detailed below for the attention of the authors.

The Abstract is insufficient in the current version. More specific information on results, particularly for the cfos experiment, is needed. Sex of the rats should be stated. The touchscreen tasks used should be clearly indicated by the typical names (i.e., TUNL and PD/RL).

The framing of the study's goal as 'developing a translational model of object-learning in the rodent' is awkward as PAL has already been thought of in this manner. Really, the authors are extending our understanding of the cortical circuitry mediating the task (and the two other tasks used) in this study. Revision of the Abstract and Introduction should re-frame the work in this manner. There are over a dozen papers using PAL in various rodent models of Alzheimer's disease that should be briefly reviewed and cited.

A justification for only using male rats should be included in the Introduction.

The last paragraph of the Intro summarizes the results. This is unnecessary and carries on too long.

Page 4, Methods. Were rats allowed to grow at a typical rate during the extended period of behavioral testing?

Page 5, Methods. When were rats killed relative to their final training session in the cfos experiment?

Page 9, Methods. The authors note that correction trials were not included in the analysis for PD and RL. Were they included in the training procedures and data analyses for the other tasks?

Page 10, Results. It should be clearly stated whether cfos expression in granular RSC was significantly higher in the dPAL group vs the CD group. From the figure, it appears that it was not.

Page 10/11, Results. References to statistical 'trends' should be removed from this section. The authors may consider using estimation statistics for some of their analyses as per eNeuro guidelines (https://www.eneuro.org/content/8/2/ENEURO.0091-21.2021).

Results. One of the great strengths of the touchscreen approach is the rich data collected from the tasks. All behavioral data including correct latencies, incorrect latencies, reward latencies, sessions to criterion, and incorrect responses should be clearly shown in the figures (or tables) and analyzed. In particular, did the lesions have an effects on latencies?

Results. The extended data should be included in the main manuscript. In particular, the OFC cfos data is interesting and should be fully considered in the context of the circuitry mediating PAL.

Results. Fig. 2B. One of the data points in the PAL group appears to be an outlier. Is this the case, and is this rat driving the effect observed?

Results. Could the lack of effect on the TUNL and PD/RL tasks simply be the result of recovery after the lesion? This possibility should be discussed.

Discussion. Critical analysis of the following issues is needed:

- Other studies have shown that performance of PAL depends on a distributed circuitry including hippocampus. Why were increases in cfos not found in hippocampus here? There are some hints of increased cfos in the dorsal hippocampus, although one outlier again seems to be driving these effects.

- Given the authors' results from the cfos experiment, does the OFC interact with the RSC during performance of PAL? Or perhaps the hippocampus?

- Neurotransmitter systems have been implicated in PAL using cholinergic, glutamatergic, and dopaminergic agents. Some discussion of how these systems in the RSC could be involved in the lesion effects observed would be useful for future researchers.

- A series of papers the authors allude to have specified a role for the retrosplenial cortex in spontaneous tests of object-place associations, most specifically the object-in-place test. These data should be considered directly, and the strengths and weaknesses of the spontaneous tests compared to the touchscreen tasks detailed.

Reviewer 2

The authors provide evidence that excitotoxic lesions of the granular RSC impaired object location memory, but left visual and spatial aspects of this memory intact. This work adds to a growing literature that suggests the RSC has a role in binding information across multiple modalities to create a cohesive episodic memory. My comments are only minor, and are listed below in no particular order.

- The focus on Alzheimer's Disease in the introduction and discussion is misleading, as the current results pertain neither to AD nor to aging, just memory and the RSC. It's a little bit of a leap given the current results to suggest a relationship to AD.

- The c-fos data could be presented in a more straightforward manner. As it stands, the graphs are difficult to interpret.

- The use of only male animals should be justified in the experimental design. It was unclear why only male rats were used here, and undermines the earlier argument regarding AD which disproportionately affects women.

- I think the discussion could expand on why the authors believe this effect is specific to the granular, rather than dysgranular, region of the RSC and lean more heavily on prior data suggesting dissociable for these regions.

## Author Response

We thank the reviewers for their insightful comments and have replied to each of them below (blue font) and revised the manuscript accordingly.

Reviewer 1 The manuscript provides the results of two experiments to assess the role of rat retrosplenial cortex in three cognitive tasks assessed in touchscreen-equipped operant conditioning chambers. Results indicated increase c-fos expression in granular retrosplenial cortex and orbitofrontal cortex following training of experienced male rats in the paired associates learning (PAL) task. Post-acquisition lesions of retrosplenial cortex also impaired performance of PAL without effects on the trial-unique non-matching-to-location task or a paired discrimination and reversal learning task. Results are considered in the context of rodent models of Alzheimer's disease.

Overall, the manuscript provides some interesting and important data regarding the role of retrosplenial cortex in cognition that will be of interest to behavioral neuroscientists. There are a number of points of concern with the manuscript in its present form. These are detailed below for the attention of the authors.

We thank the reviewer for their comments and have made the revisions outlined below accordingly.

The Abstract is insufficient in the current version. More specific information on results, particularly for the cfos experiment, is needed. Sex of the rats should be stated. The touchscreen tasks used should be clearly indicated by the typical names (i.e., TUNL and PD/RL).

We have added further detail to the abstract as suggested. Sex was already mentioned, but we have further clarified the cFos results and named the other touchscreen tasks. In addition, we have added mentioned of the sex of the subjects in several other places in the MS and added a paragraph suggesting study of sex differences as an important future direction.

The framing of the study's goal as 'developing a translational model of object-learning in the rodent' is awkward as PAL has already been thought of in this manner. Really, the authors are extending our understanding of the cortical circuitry mediating the task (and the two other tasks used) in this study. Revision of the Abstract and Introduction should re-frame the work in this manner. There are over a dozen papers using PAL in various rodent models of Alzheimer's disease that should be briefly reviewed and cited.

Well, of course tasks are always under development and subject to further validation. Nevertheless we take the reviewer's point, and therefore have shifted the framing away from "PAL as a translational tool in AD" and towards a more circuitry and RSC function-based focus throughout. We have maintained some of the mention of AD as an example as those data form much of the background support for the RSC in associative learning. We have further added discussion of papers using rodent models of AD to the introduction, as suggested:

Page 4: "To understand and develop therapies for diseases affecting cognition, including AD, animal models are essential. Given the importance of human RSC in object-location learning and success of object-location associative paradigms in human studies and the clinic, it is of considerable value to establish a translational model of object-location learning for the rodent. Touchscreen methods allow the use of such tasks in rodents that are in their most relevant aspects identical to those used in humans (Bussey et al., 2008, 1994), and, therefore, allow for validated and robust preclinical cognitive assessment of clinically relevant and highly translatable behaviours (Hvoslef-Eide et al., 2016; Palmer et al., 2021; Sullivan et al., 2021). Alongside its clinical applications, rodent PAL has shown translational validity in that it has revealed impairments in associative learning and memory in rodent models of AD (Beraldo et al., 2019; Izumi et al., 2020; Liu et al., 2024; Pang et al., 2022; Saifullah et al., 2020)." A justification for only using male rats should be included in the Introduction.

We thank the reviewer for this comment as incorporating SABV is very important in basic and translational neuroscience. We do not have any thorough justification for using only male rats beyond that these data were produced more than twelve years ago when it was, unfortunately, common practice to focus on a single sex. In lieu of justification, we have emphasized throughout the manuscript (including in the title) that the data presented are from males only, so readers of these findings do not generalize these effects across sexes. We have also added to our discussion of this as a limitation of the study:

Page 16: "One limitation of the present study is the omission of female rats and the assessment of potential sex differences. As there are known sex differences with regards to prevalence, progression, severity, and even underlying fundamental mechanisms in diseases including AD (Cui et al., 2023; Demetrius et al., 2021; Mielke, 2018), MCI (Sohn et al., 2018; Williamson et al., 2022), and in age-related cognitive deficits (Febo et al., 2020; Levine et al., 2021), understanding and appreciating the role sex plays in RSC-dependent associative learning is critical. Recent evidence using transient silencing of the anterior RSC in a classical object-location associative memory task found no sex differences (McElroy et al., 2024). However, there are anatomical and cellular sex differences in the RSC (e.g. Guma et al., 2024; Mayne et al., 2024), which could produce sex-specific effects on cognition. Therefore, the present findings using male rats only should not be generalized to females as it cannot be conclusively ascertained whether the observed effects would persist. Future experiments should strive to include both sexes and power their studies such that sex differences can be detected should they exist." The last paragraph of the Intro summarizes the results. This is unnecessary and carries on too long.

We have removed the summary of results from this paragraph.

Page 4, Methods. Were rats allowed to grow at a typical rate during the extended period of behavioral testing? Rats were food restricted to 85-90% of free-feeding baseline weight beginning 1 week after arrival and onward throughout training and testing. This is done in order to motivate the rats to perform the touchscreen tasks for food reward. This has been stated in the Methods.

Page 5, Methods. When were rats killed relative to their final training session in the cfos experiment? We thank the reviewer for catching this omission. Rats were perfused 60min following the completion of their final testing session. This detail has been added to the manuscript:

Page 5: "In Experiment 1, 60 minutes following the final session of PAL, CD training, or extended pretraining..." Page 9, Methods. The authors note that correction trials were not included in the analysis for PD and RL. Were they included in the training procedures and data analyses for the other tasks? Correction trials were included in the task and quantified, but were not counted towards the session trial limit (50) or the main accuracy score of the session. This is standard procedure for PAL and other touchscreen tasks. The purpose of CTs is to counteract side and stimulus biases, and to ensure that subjects receive a consistent number of rewards per session despite their performance on noncorrection trials. We have clarified this point in the manuscript:

Page 9: "Correction trials were quantified but were not included in session trial limit (100) nor in the session accuracy score" Page 10, Results. It should be clearly stated whether cfos expression in granular RSC was significantly higher in the dPAL group vs the CD group. From the figure, it appears that it was not.

The reviewer is correct; PAL rats had greater cFos expression than extended pretraining control rats but did not reach significance when compared to CD rats. We have clarified these results in the manuscript:

Page 10: "Expression of cFos differed between learning conditions in the granular RSC (F(2,17)=4.693, η2=0.356, p=0.0238) with cFos expression following dPAL significantly greater than in extended pretraining control rats (Cohen's d=1.763, p<0.0186; Figure 2B), but did not reach statistical significance in rats that performed CD (p=0.140)." Page 10/11, Results. References to statistical 'trends' should be removed from this section. The authors may consider using estimation statistics for some of their analyses as per eNeuro guidelines (https://www.eneuro.org/content/8/2/ENEURO.0091-21.2021).

We have removed mention of trends in this section, in Figure 2, and in the figure caption for Figure 2.

Page 10: "RSC-lesioned rats had significantly lower accuracy in the first post-lesion dPAL probe (Cohen's d=1.321, p=0.0086; session by condition interaction: F(2,36)=7.503, η2=0.0854, p=0.0019, Figure 3D; no main effects of lesion on accuracy [p=0.0566, Figure 3D] or [correction trials: p=0.0766, Figure 3E]). There were no effects on perseveration (ps>0.177; Figure 3F)." Results. One of the great strengths of the touchscreen approach is the rich data collected from the tasks. All behavioral data including correct latencies, incorrect latencies, reward latencies, sessions to criterion, and incorrect responses should be clearly shown in the figures (or tables) and analyzed. In particular, did the lesions have an effects on latencies? These secondary measures are indeed a strength of the touchscreen approach. When these data were collected (over 12 years ago), the primary measures (sessions to criterion, correct responses, correction trials) were extracted for analysis, and, at that time, many of the secondary measures were not. While some of the original database files still exist, many have been lost in lab moves and in various hardware updates. As such, we do not have many of the secondary measures, including latencies, for this set of experiments.

Results. The extended data should be included in the main manuscript. In particular, the OFC cfos data is interesting and should be fully considered in the context of the circuitry mediating PAL.

We have included the extended data pertaining to cFos expression in Figure 2 and have expanded upon the discussion of the OFC (and dCA1/2 and lateral CPu) and the circuitry mediating PAL in the discussion, as requested below.

Page 13: "First, we found the rat retrosplenial cortex showed increased cFos expression (a proxy for neural activation) following object-location paired-associates learning, suggesting it is actively recruited during performance of the task. The lateral caudate-putamen and orbitofrontal cortex also exhibited increased cFos expression during PAL performance. PAL has been shown to be sensitive to lesions of the dorsal striatum in the mouse (Delotterie et al., 2015). The orbitofrontal cortex (Hall-McMaster et al., 2017; Schoenbaum and Roesch, 2005) and striatum (Avvisati et al., 2024; Garr, 2017; Liljeholm and O'Doherty, 2012; Stelly et al., 2020; Yin and Knowlton, 2004) have both been implicated in associative learning and memory more generally. There is some evidence of functional connectivity between RSC and both regions (Monko and Heilbronner, 2021), but whether the observed concurrent activation of these regions during PAL results from direct interactions between the regions remains to be determined. Interestingly, despite many reports demonstrating hippocampal function as essential to rodent PAL (Al-Onaizi et al., 2017; Delotterie et al., 2015; Kim et al., 2015, 2016; Talpos et al., 2009), the only increase in cFos expression we observed was in rats who performed CD compared to extended pretraining controls. This lack of clear hippocampal involvement in PAL may be a result of the extent of training on PAL prior to tissue collection. One model of RSC-hippocampal interaction during learning (Miller et al., 2014) places RSC in a dual role: 1) RSC outputs to the hippocampus information about cues and context and 2) is the target of hippocampal output during systems-level consolidation. In this way, following extensive associative learning, it is possible that the performance of a task will have significantly reduced hippocampal involvement or even become hippocampal-independent, as has been previously demonstrated in a flavour-location paired-associates task (Tse et al., 2007). " Results. Fig. 2B. One of the data points in the PAL group appears to be an outlier. Is this the case, and is this rat driving the effect observed? We thank the reviewer for pointing this out. We were initially not removing any outliers in our analyses because they occurred sporadically throughout the data, suggesting real data and not just methodological issues. However, when we considered the hippocampal data and possible outlier that this reviewer mentions below, we decided to remove it as that outlier was driving an increase in variability in that group and obscuring important effects.

We have now removed outliers using the ROUT method:

Page 9: "Statistical analyses were performed using GraphPad Prism (Version 9.1.0, San Diego, CA, USA). IEG expression was assessed with one-way ANOVAs with Tukey post hocs and the behaviour condition (dPAL, CD, or control) as the between-subjects factor, with outliers removed using the ROUT method (Q=1%) and Brown-Forsythe ANOVAs employed when standard deviations were unequal." This resulted in a few changes to the findings. Importantly, the granular RSC conclusions did not change; however, the dorsal CA1/2 and lateral CPu showed main effects that we subsequently analyzed:

Page 10: "The dorsal CA1/2 (F(2,17)=4.366, η2=0.339, p=0.0295; Figure 2D), orbitofrontal cortex (F(2,18)=11.55, η2=0.562, p=0.0006; Figure 2F), and lateral caudate-putamen (F(2,17)=4.249, η2=0.333, p=0.0319; Figure 2G) showed changes in cFos expression following learning. In the dorsal CA1/2, CD learning resulted in greater cFos expression than in control rats (Cohen's d=1.362, p=0.0256; Figure 2D). In the orbitofrontal cortex, dPAL learning resulted in greater cFos expression than CD (Cohen's d=1.544, p=0.0128; Figure 2F) and control rats (Cohen's d=2.677, p=0.0004; Figure 2F). In the lateral caudate-putamen, dPAL learning resulted in greater cFos expression than CD (Cohen's d=2.326, p=0.025; Figure 2G). The dysgranular RSC did not exhibit changes in cFos expression (p=0.647; Figure 2C) nor did other dorsal (ps>0.0566; Figure 2D) or ventral (ps>0.150; Figure 2E) hippocampal, cortical (ps>0.221; Figure 2F), or striatal (ps>0.429; Figure 2G) regions, nor the subiculum (p=0.306; Figure 2H)." Results. Could the lack of effect on the TUNL and PD/RL tasks simply be the result of recovery after the lesion? This possibility should be discussed.

We have added discussion of this to the manuscript:

Page 13-14: "The RSC lesions were still present and complete following the end of the testing battery (see Extended Data, Figure 3-1). While in some cases compensatory mechanisms can lead to recovery of function following brain damage, the lesions in the present study were still functionally effective through PVD testing, as rats with RSC lesions performed significantly more correction trials than controls during this task." Discussion. Critical analysis of the following issues is needed:

- Other studies have shown that performance of PAL depends on a distributed circuitry including hippocampus. Why were increases in cfos not found in hippocampus here? There are some hints of increased cfos in the dorsal hippocampus, although one outlier again seems to be driving these effects.

- Given the authors' results from the cfos experiment, does the OFC interact with the RSC during performance of PAL? Or perhaps the hippocampus? As these comments are linked, we'll address them together. Following outlier removal (mentioned above) we found not only activation of OFC, but also changes in dCA1/2 and lateral CPu with touchscreen training. We have added discussion of the OFC, dCA1/2, and lateral CPu (see previous response or Page 13 of the manuscript).

- Neurotransmitter systems have been implicated in PAL using cholinergic, glutamatergic, and dopaminergic agents. Some discussion of how these systems in the RSC could be involved in the lesion effects observed would be useful for future researchers.

We have added this as an important future direction in the discussion:

Page 15-16: "Future studies should also explore the mechanistic roles played by RSC in associative learning and memory. For instance, as glutamate, acetylcholine, and dopamine neurotransmitter systems have previously been implicated in PAL (Bartko et al., 2011; Bethus et al., 2010; Day et al., 2003; Harel et al., 2013; Lins and Howland, 2016; Talpos et al., 2009), and there is evidence of each of these systems within RSC (Corcoran et al., 2011; de Landeta et al., 2022; Todd et al., 2019; Vallianatou et al., 2019), it would be advantageous in future studies to explore the neurotransmitters involved in the role of RSC in associative learning." - A series of papers the authors allude to have specified a role for the retrosplenial cortex in spontaneous tests of object-place associations, most specifically the object-in-place test. These data should be considered directly, and the strengths and weaknesses of the spontaneous tests compared to the touchscreen tasks detailed.

We have added to the discussion of the previous studies of multimodal associative learning, highlighting the differences of using multiple testing modalities as overall strengths, and highlighting the scientific opportunities afforded by these tasks:

Page 14: "Critically, the convergence of these data from markedly different associative learning tasks - some appetitive, some aversive, various stimulus modalities throughout - is highly suggestive of a consistent role of the RSC in associative learning. With respect to testing object-in-place associative learning and memory, these different approaches confer different advantages. Touchscreen PAL confers a number of advantages, including a high degree of standardization, robust translational and construct validity, high-throughput data collection, and minimal experimenter involvement during testing. Spontaneous tasks in open fields confer different advantages; for example, usable data gathered from a low number of trials, and a sample-delay-choice structure that allows for the isolation of memory processes such as encoding, consolidation, and retrieval (such as in McElroy et al., 2024)." With regards to the comparison of spatial working memory tasks, we have added the following to compare the classical tasks with touchscreen methods in order to highlight the strengths and differences in these approaches:

Page 16: "In this way, the touchscreens allow for more selective testing of spatial working memory using TUNL by removing or reducing the salience of cue-context or proximal-distal association. However, classical tests are still very useful for the evaluation and understanding of the use of allocentric, egocentric, intra-maze, and extra-maze cues in task performance as they can be experimentally manipulated, and, as such, have contributed much to our evolving understanding of the role of the RSC and its divisions in spatial memory." - Reviewer 2 The authors provide evidence that excitotoxic lesions of the granular RSC impaired object location memory, but left visual and spatial aspects of this memory intact. This work adds to a growing literature that suggests the RSC has a role in binding information across multiple modalities to create a cohesive episodic memory. My comments are only minor, and are listed below in no particular order.

We thank the reviewer for their comments and have made the revisions outlined below accordingly.

- The focus on Alzheimer's Disease in the introduction and discussion is misleading, as the current results pertain neither to AD nor to aging, just memory and the RSC. It's a little bit of a leap given the current results to suggest a relationship to AD.

We have clarified throughout that the focus of the paper is on the RSC's involvement in associative learning. We maintain AD as an example of disrupted RSC function but have been sure to clearly disambiguate the current study from the implication that it is a model of AD.

- The c-fos data could be presented in a more straightforward manner. As it stands, the graphs are difficult to interpret.

We have rearranged the training groups so that they correspond better to the writing of the manuscript (control first, then CD, then PAL). We have also included the former Extended Data Figure 2-1 in Figure 2 as suggested by reviewer 1 for a broader representation of the cFos data throughout the regions analyzed.

- The use of only male animals should be justified in the experimental design. It was unclear why only male rats were used here, and undermines the earlier argument regarding AD which disproportionately affects women.

We thank the reviewer for this comment as incorporating SABV is very important in basic and translational neuroscience. We do not have any thorough justification for using only male rats beyond that these data were produced more than twelve years ago when it was, unfortunately, common practice to exclude females. In lieu of justification, we have emphasized throughout the manuscript (including in the title) that the data presented are from males only, so readers of these findings do not generalize these effects across sexes. We have also throughout the manuscript adjusted the focus of the paper away from AD and development of a translational task targeting AD, as suggested above. We have also added to our discussion of this as a limitation of the study:

Page 16: "One limitation of the present study is the omission of female rats and the assessment of potential sex differences. As there are known sex differences with regards to prevalence, progression, severity, and even underlying fundamental mechanisms in diseases including AD (Cui et al., 2023; Demetrius et al., 2021; Mielke, 2018), MCI (Sohn et al., 2018; Williamson et al., 2022), and in age-related cognitive deficits (Febo et al., 2020; Levine et al., 2021), understanding and appreciating the role sex plays in RSC-dependent associative learning is critical. Recent evidence using transient silencing of the anterior RSC in a classical object-location associative memory task found no sex differences (McElroy et al., 2024). However, there are anatomical and cellular sex differences in the RSC (e.g. Guma et al., 2024; Mayne et al., 2024), which could produce sex-specific effects on cognition. Therefore, the present findings using male rats only should not be generalized to females as it cannot be conclusively ascertained whether the observed effects would persist. Future experiments should strive to include both sexes and power their studies such that sex differences can be detected should they exist." - I think the discussion could expand on why the authors believe this effect is specific to the granular, rather than dysgranular, region of the RSC and lean more heavily on prior data suggesting dissociable for these regions.

We have added to our discussion section on the dissociable nature of the RSC regions, concluding with:

Page 15: "When comparing granular and dysgranular RSC, overall, granular RSC receives much hippocampal and parahippocampal information (spatial and contextual) whereas dygranular RSC receives visual input, with extensive cross-talk between the regions (Aggleton et al., 2021). As considerable research has shown the hippocampus to be involved in rodent PAL (Al-Onaizi et al., 2017; Delotterie et al., 2015; Kim et al., 2015, 2016; Talpos et al., 2009), it is not surprising that granular RSC specifically would be involved in touchscreen PAL, with less involvement of dysgranular RSC as the touchscreen chamber creates an overall less visually rich environment than in many conventional tasks."
