# Supplementary figures and images for: The Granular Retrosplenial Cortex Is Necessary in Male Rats for Object-Location Associative Learning and Memory, But Not Spatial Working Memory or Visual Discrimination and Reversal, in the Touchscreen Operant Chamber
Source: eNeuro. 2024 Jun 14;11(6):ENEURO.0120-24.2024. doi: 10.1523/ENEURO.0120-24.2024 (PMC11208985; doi:10.1523/ENEURO.0120-24.2024)

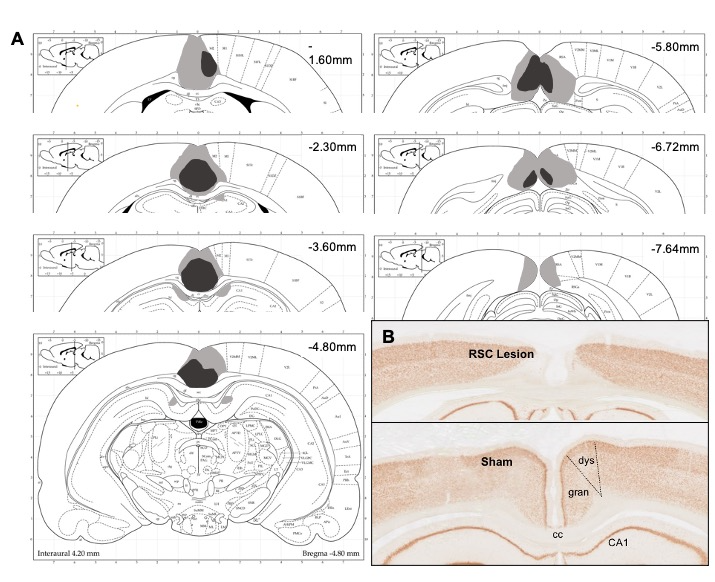

Supplement: Figure 3-1 — Extent of retrosplenial cortex lesions. A) Representation of the maximum (light grey) and minimum (dark grey) lesioned regions. Adapted from (Paxinos and Watson, 2006). B) Representative images of RSC lesion (upper) and sham lesion (lower) tissue. Lesions were highly specific to the granular RSC leaving the dysgranular RSC largely spared. Download Figure 3-1, TIF file. [file eneuro-11-ENEURO.0120-24.2024-s002.tif]
